# Hormone profiling, WHO 2010 grading, and AJCC/UICC staging in pancreatic neuroendocrine tumor behavior

**DOI:** 10.1002/cam4.96

**Published:** 2013-08-06

**Authors:** Emilie Morin, Sonia Cheng, Ozgur Mete, Stefano Serra, Paula B Araujo, Sara Temple, Sean Cleary, Steven Gallinger, Paul D Greig, Ian McGilvray, Alice Wei, Sylvia L Asa, Shereen Ezzat

**Affiliations:** 1 Department of Medicine, Princess Margaret Cancer Centre, University Health NetworkToronto, Ontario, Canada; 2 Department of Pathology, Princess Margaret Cancer Centre, University Health NetworkToronto, Ontario, Canada; 3 Department of Surgery, Princess Margaret Cancer Centre, University Health NetworkToronto, Ontario, Canada

**Keywords:** Carcinoid, gastrinoma, insulinoma, neuroendocrine tumors, pNET

## Abstract

Pancreatic neuroendocrine tumors (pNETs) are the second most common pancreatic neoplasms, exhibiting a complex spectrum of clinical behaviors. To examine the clinico-pathological characteristics associated with long-term prognosis we reviewed 119 patients with pNETs treated in a tertiary referral center using the WHO 2010 grading and the American Joint Committee on Cancer/International Union Against Cancer (AJCC/UICC) staging systems, with a median follow-up of 38 months. Tumor size, immunohistochemistry (IHC) profiling and patient characteristics-determining stage were analyzed. Primary clinical outcomes were disease progression or death. The mean age at presentation was 52 years; 55% were female patients, 11% were associated with MEN1 (multiple endocrine neoplasia 1) or VHL (Von Hippel–Lindau); mean tumor diameter was 3.3 cm (standard deviation, SD) (2.92). The clinical presentation was incidental in 39% with endocrine hypersecretion syndromes in only 24% of cases. Nevertheless, endocrine hormone tissue immunoreactivity was identified in 67 (56.3%) cases. According to WHO 2010 grading, 50 (42%), 38 (31.9%), and 3 (2.5%) of tumors were low grade (G1), intermediate grade (G2), and high grade (G3), respectively. Disease progression occurred more frequently in higher WHO grades (G1: 6%, G2: 10.5%, G3: 67%, *P* = 0.026) and in more advanced AJCC stages (I: 2%, IV: 63%, *P* = 0.033). Shorter progression free survival (PFS) was noted in higher grades (G3 vs. G2; 21 vs. 144 months; *P* = 0.015) and in more advanced AJCC stages (stage I: 218 months, IV: 24 months, *P* < 0.001). Liver involvement (20 vs. 173 months, *P* < 0.001) or histologically positive lymph nodes (33 vs. 208 months, *P* < 0.001) were independently associated with shorter PFS. Conversely, tissue endocrine hormone immunoreactivity, independent of circulating levels was significantly associated with less aggressive disease. Age, gender, number of primary tumors, and heredity were not significantly associated with prognosis. Although the AJCC staging and WHO 2010 grading systems are useful in predicting disease progression, tissue endocrine hormone profiling provides additional information of potentially important prognostic value.

Although the AJCC staging and WHO 2010 grading systems are useful in predicting disease progression, tissue endocrine hormone profiling provides additional information of potentially important prognostic value.

## Introduction

Neuroendocrine tumors (NETs) represent a challenge in endocrine oncology. For many years, physicians considered NETs biologically “benign” neoplasms in the absence of progressive metastatic disease. However, other than pancreatic neuroendocrine microadenomas, which are defined as pancreatic neuroendocrine proliferations that measure less than 0.5 cm, retrospective data suggested that all pancreatic NETs (pNETs) are malignant neoplasms 1.

Facing the wide biological spectrum of pNETs, clinicians, and pathologists have attempted to identify prognostic factors that would aid management decisions. Proliferative indices have emerged as useful prognostic markers in determining disease progression and possibly overall survival in pNETs 2–4. Nevertheless, there are several outstanding controversies surrounding the ideal terminology and staging systems that should be applied for NETs. In 2010, the WHO classification introduced a system of nomenclature that combined the differentiation and grading features of gastroenteropancreatic (GEP) NETs. The three tiers include integration of the mitotic count (Grade 1: <2 mitoses/10 HPF (high-power field), Grade 2: 2–20 mitoses/10 HPF, and Grade 3: >20 mitoses/10 HPF) and the Ki-67 (MIB-1) labelling index (Grade 1: <3%, Grade 2: 3–20%, Grade 3: >20%) to better classify the biological aggressiveness of these neoplasms 5. The two TNM/staging systems proposed by the American Joint Committee on Cancer/International Union Against Cancer (AJCC/UICC) 6 and the European Neuroendocrine Tumor Society (ENETS) 7 also differ in their definitions of stage groupings. The 7th edition AJCC/UICC TNM staging system introduced a site-specific and grade-dependent staging model for GEP-NETs 6. The AJCC/UICC recommended that all grades of pNETs should be staged using the TNM criteria used for ductal adenocarcinoma of the pancreas. This model distinguishes localized pNETs (stage I), locally advanced resectable pNETs (stage II), locally advanced unresectable pNETs (stage III), and pNETs with distant metastases (stage IV). The prognostic validity of TNM has been tested in assessing overall survival (OS) 3,8,9. Studies examining other potential prognostic factors have been limited by small sample sizes mainly due to the relative rarity of these tumors.

Although both the WHO grading and AJCC staging systems have been introduced relatively recently, there remains a need to validate the prognostic impact of these classification systems in large groups of patients with a diverse spectrum of presentations. Therefore, we aimed to compare the prognostic impact of the WHO 2010 grading and AJCC/UICC staging systems in pNETs in a large retrospective analysis of clinico-pathological parameters at diagnosis and clinical outcomes. We also assessed long-term follow-up in selected groups who underwent biotherapy with somatostatin analogs.

## Patients and Methods

### Patient population

This retrospective study evaluated the clinical data and the treatment outcomes of 119 patients diagnosed between 1979 and 2011, with histologically confirmed pNETs diagnosed at the University Health Network (UHN), a tertiary referral center for the management of neuroendocrine tumors in Toronto, Canada. UHN Institutional Research Ethics Board approval was obtained for the study. Written informed consent was provided for data collection at the time of surgery. All values shown in the figures represent actual N-values with corresponding percentages, means or medians depicted in the text.

### Endocrine evaluations

Circulating biomarkers were performed following an overnight fast including serum levels of glucose, chromogranin-A, insulin, gastrin, glucagon, pancreatic polypeptide, and where clinically indicated vasoactive intestinal peptide (VIP).

### Definition of functionality

In addition to the standard panel of markers of neuroendocrine differentiation (synaptophysin, chromogranin, and neuron-specific enolase), immunohistochemistry (IHC) included staining for insulin, glucagon, somatostatin, pancreatic polypeptide, gastrin, and VIP. If symptoms and circulating levels attributable to the corresponding peptide were concordant with immunostaining, the tumors were classified as clinically functional.

### WHO tumor grading

Grading was performed following the WHO 2010 classification 1,5 that separates tumors according to their proliferative rates as follows: G1 (low grade): <2 mitoses/10 HPF and/or <3% Ki67 index, G2 (intermediate grade): 2–20 mitoses/10 HPF and/or 3–20% Ki67 index, G3 (high grade): >20 mitoses/10 HPF and/or >20% Ki67 index. In instances where the mitotic count and the Ki67 labelling index provided conflicting information, the higher value was adopted for grading purposes.

### AJCC/UICC TNM staging

For staging, we used the 7th edition of the AJCC/UICC TNM staging system designated for pNETs 10. Tumor staging was performed combining information from radiographic modalities including CT-scanning or MR imaging, as well as comprehensive data from the pathology report. Other imaging modalities such as nuclear bone scans and octreo-scanning were also used to determine extent of metastatic disease.

### Assessment of clinical outcomes

Progression free survival (PFS) was defined as the number of months from the date of surgery (or time of the first diagnostic imaging study in those without surgery) to the first documentation of disease progression. Disease stability was determined on the basis of objective imaging studies confirming absence of tumor progression or recurrence. OS was defined as the number of months from the date of surgery to the date of the last follow-up visit or time of death. If the patient did not have surgery, the initial date was defined by the first diagnostic imaging study. Any missing information on final outcome was treated as death on a “worst-case scenario” basis for an intent-to-treat analysis. Even though AJCC staging was initially validated to assess survival, the evaluation of intermediate end-points such as PFS is of relevance in order to define therapeutic strategies and prognosis; we analyzed PFS with this staging system as an exploratory maneuver.

### Statistical analyses

Statistical analysis was performed using Excel 14.2.1 software for databases and IBM/SPSS version 20 for analysis. All variables were reported according to their distribution by means, median, standard errors (SE) or deviations (SD), variance, minimum, maximum or range as indicated, and their frequencies as proportions (%). We used *t*-tests to compare means or Mann–Whitney *U*-test according to variable distribution. We performed analysis of survival with Kaplan–Meier curves and comparisons between factors and strata when necessary. For comparisons in survival analysis we used generalized Wilcoxon test between factors. Finally, we modelled multinomial logistic analyses to evaluate the combined contribution of variables. For the biotherapy section, we included an intention-to-treat analysis 11. Statistical significance was considered reached when *P*-values were below 0.05.

## Results

### Patient characteristics

Of the 119 patients, 66 were female patients (55.5%), 11 (9%) had MEN1 (multiple endocrineneoplasia 1), and 2 (2%) had VHL (Von Hippel–Lindau). Other cancers were documented in 22 patients (19%), the most common being thyroid cancer, which was diagnosed in six patients (5%). Nearly half (*n* = 53; 44.5%) of patients were diagnosed with functioning tumors of which 31 (26.1%) were insulinomas while 46 (38.7%) were diagnosed as nonfunctional. The mean follow-up was 38 months (1–360 months). Detailed patient characteristics are shown in Table 1.

**Table:** Patient characteristics

Patients characteristics		Number		%
Age (years)		52 (mean)		
Gender	Male	53		45
	Female	66		55
Genetic	MEN1	11		9
	VHL	2		2
Other cancer		22		19
Clinical presentation				
Incidental finding		46		39
Endocrine hypersecretion^1^		28		24
Abdominal pain		20		17
Gastrointestinal dysmotility		6		5
Pancreatic or liver enzyme abnormality		5		4
Endocrine evaluation				
Functional		53		44.5
Nonfunctional		64		55
Elevated serum chromogranin A level		5/23		22
Radiology				
Octreoscan avidity		20/39		51
Tumor diameter		33		
AJCC/UICC stage 11, 5 (mm)				
I		46		39
IIA		26		22
IIB		18		15
IIIB		10		8
IV		19		16
WHO 2010 grade				
G1		50		42
G2		38		31.92
G3		3		32.5
Missing		28		23.5
Hormone immunostaining		IHC+		%
Not assessed		9		7.6
Insulin		30		25.2
Glucagon		17		14.3
Vasoactive intestinal peptide		9		7.68
Gastrin		2		1.72
Somatostatin		1		0.81
Other peptides		51		42.9
Treatment strategy				
Resection of primary tumor		109		92
Adjuvant octreotide		17		14
Hepatic embolization		7		6

Clinical presentation corresponding to a recognizable endocrine hypersecretion syndrome, MEN1, multiple endocrine neoplasia 1; VHL, Von Hippel–Lindau; IHC, immunohistochemistry.

### Primary tumor size, WHO grade, and AJCC/UICC stage

**Figure 1:**
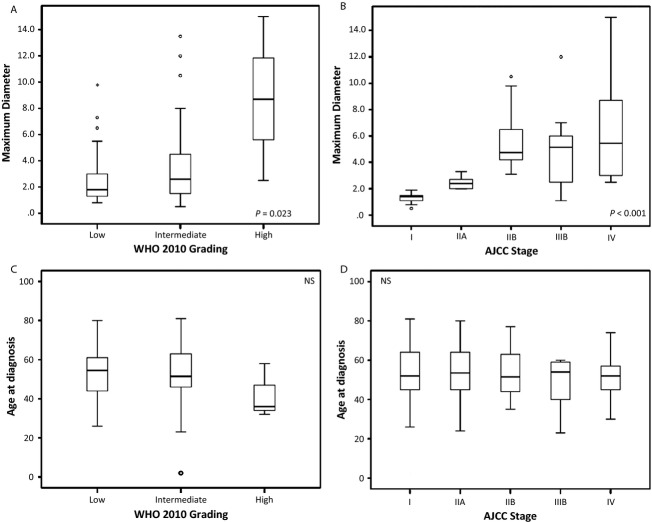
Relationship between primary pancreatic neuroendocrine tumor size and age with WHO grade and AJCC Stage. (A) Maximum tumor diameter in cm was stratified according to WHO grade. The median for G1 tumors was 1.8, for G2 2.6, for G3 8.7 (*P* = 0.023). (B) Maximum tumor diameter was stratified according to AJCC stage, resulting in a median for stage I of 1.4, 2.4 for stage IIA, 4.7 for stage IIB, 5.1 for stage IIIB, and 5.45 for stage IV (*P* < 0.001). (C) Age at diagnosis according to WHO grade with a G1 median of 54 years, 51 for G2, and 36 for G3 (NS). (D) Age diagnosis stratified by AJCC stage show a median of 52 years for stage I, 53.06 for IIA, 51.5 for IIB, 54.0 for IIIB, and 52 for stage IV (NS). (Low grade [G1], intermediate grade [G2], high grade [G3]).

The size of the primary tumor increased gradually with the WHO grades; median for G1 was 1.8 cm (variance 3.9), for G2 was 2.6 cm (9.4), for G3 was 8.7 cm (39.0); *P* = 0.023 (Fig. 1A). Similarly, for AJCC Stage, median for stage I was 1.4 cm (variance 0.96), 2.4 cm for stage IIA (0.168), 4.7 cm for stage IIB (4.30), 5.1 cm for stage III (10.52), and 5.45 cm for stage IV (17.68); *P* < 0.001 (Fig. 1B). This was confirmed by ANOVA, where the means (SD) were 1.31 (0.3) for stage I, 2.42 (0.41) for stage IIA, 5.5 (2.0) for stage IIB, 4.89 (3.24) for stage III, and 6.6 (4.2) for stage IV (*P* < 0.001). Age at time of diagnosis did not correlate with tumor grade (Fig. 1C) or with stage (Fig. 1D).

### Disease stability or progression

The majority of patients showed disease stability (*n* = 83; 69.7%), death related to disease occurred in three cases (2.5%). However, the final outcome was not known in 21 cases (17.6%), which were assigned on an intent-to-treat analysis to the death outcome.

#### Progression according to WHO 2010 grade

**Figure 2:**
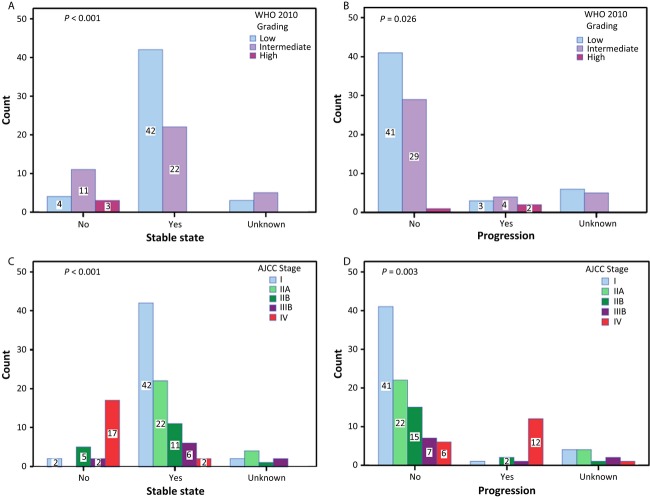
Pancreatic NET disease stability by WHO grade and AJCC stage. (A) Comparison of disease stability according to WHO grade. Stable disease was noted in 42/49 G1, 22/38 G2, and 0/3 G3 (*P* < 0.001). (B) Progression of disease was stratified according to WHO grade. Disease progression was noted in 3/50 G1 tumors, 4/38 G2, and 2/3 G3 (*P* = 0.026). (C) Disease stability according to AJCC staging as present in 42/46 stage I patients, 22/26 stage IIA, 11/17 stage IIB, 6/10 stage IIIB, and 2/19 stage IV (*P* < 0.001). (D) Progression according to AJCC is shown. Accordingly, 1/46 stage I cases, 0/26 stage IIA, 2/18 stage IIB, 1/10 stage IIIB, and 12/19 stage IV (*P* = 0.033).

While 85.7% and 57.9% of G1 and G2 tumors remained stable, none of the G3 pNETs showed stability (*P* < 0.001) (Fig. 2A). Conversely, disease progression correlated positively with the WHO tumor grade where only 6% of G1 tumors progressed compared with 10.5% of G2, and 100% of G3 pNETs (*P* = 0.026) (Fig. 2B).

#### Progression according to AJCC/UICC stage

Disease stability inversely correlated with the AJCC/UICC stage (Fig. 2C); 91.3% of stage I, 84% of stage IIA, 64.7% of stage IIB, 60% of stage IIIB, and 10.5% of stage IV patients remained stable (*P* < 0.001). Conversely, disease progression positively correlated with the AJCC/UICC stages where only 1/46 stage I patients showed disease progression, 0/26 stage IIA (0%), 2/18 stage IIB (11.1%%), 1/10 stage IIIB (10%), and 12/19 stage IV (63.2%); overall comparison, *P* = 0.033 (Fig. 2D).

### Progression free survival

The overall mean estimated PFS was 145.36 months (SE 28.18) [95% CI: 90.1–200.6]. Of those with recurrences, 10 were loco-regional, and 18 developed distant metastases.

#### PFS according to WHO 2010 grade

**Figure 3:**
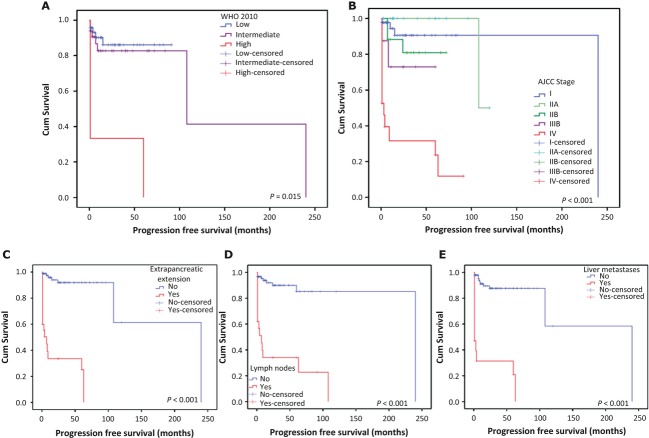
Progression free survival (PFS) analyses. (A) Kaplan–Meier survival analyses of PFS according to WHO grade. PFS estimates were 79.3 months for G1 (Ne = 39.5), 144.7 months for G2 (Ne = 29.5), and 20.6 months for G3 (Ne = 3) (*P* = 0.015). (B) Stratified by AJCC staging, PFS estimate was 218.3 months for stage I (Ne = 37), 114.0 months for stage IIA (Ne = 20.5), 60.8 months for stage IIB (Ne = 16.0), 45.0 months for stage IIIB (Ne = 7.5), and 24.6 months for stage IV (Ne = 17.5) (*P* < 0.001). (C) Extra-pancreatic extension (Ne = 23) results in a PFS estimate of 22.9 months versus 181.3 months for cases without it (Ne = 75.5) (*P* < 0.001). (D) The presence of lymph nodes shows a similar result with a PFS estimate of 33.8 months for positive (Ne = 20) versus 208.3 months for negative nodes (Ne = 78.5) (*P* < 0.001). (E) The presence of liver metastases (Ne = 15.5) was associated to a PFS of 20.5 months versus 173.0 months without metastases (Ne = 83.0) (*P* < 0.001). Ne, Number exposed to risk.

The PFS estimates trended to diminish with WHO tumor grades, which were 79.3 (SE 4.9) months for G1, 144.7 (SE 43.6) months for G2, and 20.6 (SE 19.6) months for G3 (*P* = 0.015) (Fig. 3A).

#### PFS according to AJCC/UICC stage

The PFS estimates were more clearly associated with AJCC/UICC stages, which were 218.3 months (SE 14.0) for stage I, reduced to 114.0 months (SE 4.2) for stage IIA, 60.8 months (SE 5.8) for stage IIB, 45.0 months (SE 9.0) for stage IIIB, and 24.6 months (SE 8.6) for stage IV (*P* < 0.001) (Fig. 3B).

#### PFS according to extra-pancreatic extension

The presence of extra-pancreatic extension was associated with significantly shorter PFS estimates of 22.9 months (SE 6.3) versus 181.3 months (SE 36.3) for cases without extra-pancreatic extension (*P* < 0.001) (Fig. 3C). Similarly, the presence of lymph node or liver metastases was associated with shorter PFS (each, *P* < 0.001); lymph node involvement sharply reduced the PFS from 208.3 months (SE 12.3) to 33.8 months (SE 11.0) (Fig. 3D), while liver metastasis reduced the PFS from 173.0 months (SE 33.8) to 20.5 months (SE 7.6) (Fig. 3E).

#### PFS according to endocrine functionality

Although the PFS estimates of clinically functioning pNETs (144.7 months; SE 30.9) were longer than clinically nonfunctioning pNETS (77.8 months, SE 30.9); this difference did not reach statistical significance. Moreover, the status of immunohistochemical positivity for hormones in clinically functioning and nonfunctioning pNETs failed to show a statistical correlation with PFS estimates.

### Overall survival

Three deaths were confirmed and 21 cases were lost to follow-up. This allowed for two survival scenario analyses: best-case scenario with three deaths and worst-case scenario (intent-to-treat) with 24 deaths. Mean OS was 225.3 months (SE 8.8) (95% CI: 207.9–242.6). By WHO tumor grade, the mean estimated OS was 90.0 months for G1, 200.0 months for G2, 60.0 months for G3; non-significant (NS). The intent-to-treat analysis yielded an overall mean OS of 195.3 months (SE 11.1) (95% CI 173.4–217.1). The mean estimated OS was not significantly influenced by the WHO grade, which ranged from 77.0 months (SE 5.5) in G1 to 40.3 months (SE 16.0) in G3 pNETs; NS.

In contrast to the WHO grade, the AJCC/UICC stages yielded statistically significant OS estimates (*P* = 0.001), which were distributed as follows: 217.5 months (SE 10.7) in stage I, 113.3 months (SE 6.3) in stage IIA, 64.0 months (SE 5.1) in stage IIB, 45.0 months (SE 9.0) in stage IIIB, and 49.0 months (SE 12.7) in stage IV.

### Observations on biotherapy

To further examine the utility of grading and staging in monitoring pNET behavior, we examined the performance of these measures in patients receiving somatostatin analog therapy. As this therapy antedated more recent evidence of their benefits in NETs, this treatment was offered mainly to patients with hormone hypersecretion. With this in mind, the proportion of patients receiving adjuvant octreotide therapy was 15/17 in clinically aggressive cases (88.2%) versus 2/17 [11.8%], *P* = 0.011). As such, disease stability was less frequent in those receiving octreotide (3/17; 17.6%) compared to those who did not receive the medication (80/101; 79.2%), *P* < 0.001. Using the WHO grades, the mean estimated PFS for the octreotide-treated group was 7 months (SE 0) versus 81.5 months (SE 4.6); estimated PFS for those not offered octreotide (*P* = 0.025) for G1; 29.1 (SE 13.8) months versus 165.7 (SE 55.1) months for G2 (*P* < 0.001), and 1 (SE 0) month versus 30.5 (SE 29.5) months (NS) for G3. The OS by intent-to-treat analysis according to the WHO grade revealed no significant differences in comparisons within the non octreotide-treated cases. The OS by intent-to-treat analysis with respect to the AJCC stages did not show a significant difference in the octreotide-treated cases. For the non octreotide-treated cases, the overall comparison was statistically significant with advancing stage (*P* = 0.012). Specifically, OS in the non octreotide-treated group ranged from 240.0 months for stage I; 120 months for stage IIB; 60 months for stage IIIB, to 80 months for stage IV.

### Immunohistochemical markers

**Figure 4:**
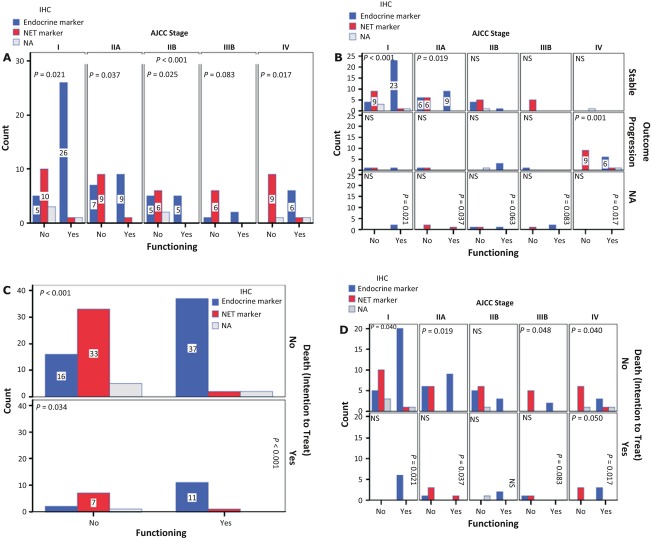
Protein tissue markers by IHC. Endocrine markers and NET markers are shown according to the following: (A) AJCC stage and clinical functioning status, showing significantly greater proportions of endocrine markers in earlier AJCC stages, but greater proportions of NET markers alone in more advanced stages (overall comparison: *P* < 0.001). (B) Clinical outcome, clinical functional status, and AJCC stage: lower stage, stable tumors showed a significantly higher proportion of endocrine markers (*P* < 0.001 and *P* = 0.019) whereas stage IV, progressing tumors showed a significantly higher proportion of NET markers alone (*P* = 0.001). (C) Death (intent-to-treat) and clinical functional status: endocrine markers showed a significantly higher proportion in functional tumors (41.0% vs. 2.6%, *P* < 0.001); in the demised group the proportion of endocrine markers in the nonfunctioning group is reduced (16.8% vs. 9.1%). (D) Death (intent-to-treat), clinical functional status and AJCC stage: Endocrine markers displayed significantly higher proportions in early stages (AJCC I: 62.5% vs. 27.5%, *P* = 0.040), whereas the proportion of NET markers alone was higher in more advanced stages (AJCC IV: 58.3% vs. 25%, *P* = 0.040); in the demised group, the proportions favored NET markers from early stages (AJCC IIA: 60% vs. 20%), but the small N precludes statistical interpretation.

**Figure 5:**
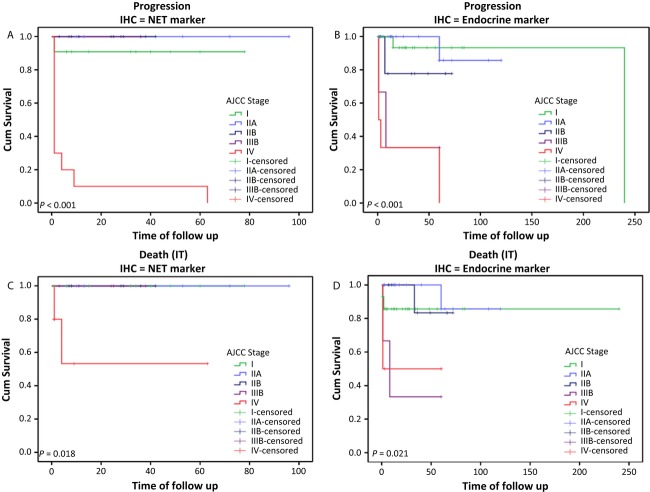
Kaplan–Meier survival curves for time to progression according to AJCC stage and, (A) presence of NET marker, displaying a significant difference across stages (*P* < 0.001) or (B) presence of endocrine marker, also significantly different (*P* < 0.001). Kaplan–Meier survival curves for overall survival (intention-to-treat) according to AJCC stage and (C) presence of NET markers show significant difference between AJCC stages (*P* = 0.018) or (D) presence of endocrine markers, display a significant difference between AJCC stages (*P* = 0.021).

Of 119 cases, 110 tumors had complete immunohistochemical assessment. Of these, 91 cases permitted complete assignment of WHO grading. For analytical purposes, we grouped markers into NET markers which included the following: Chromogranin A, synaptophysin, CK19, CD56, and CD99. Endocrine hormone markers included the following: insulin, glucagon, VIP, somatostatin, gastrin, pancreatic polypeptide. Endocrine hormone markers were present in 67 (56.3%) cases while NET markers alone without hormone staining was noted in 43 (36.1%), IHC was not determined in nine (7.6%) cases. Of note, 18 (15.4%) cases showing endocrine markers were clinically nonfunctional. Moreover, cases with only NET markers were significantly associated with higher AJCC stages and poorer outcomes (Figs. 4 and 5).

Multinomial logistic modelling identified a significant contribution from AJCC stage, WHO grade, Ki-67, and octreoscan positivity. Moreover, NET markers were over represented in higher stage tumors while insulin and other endocrine differentiation markers were more represented in lower stage tumors. Specifically, AJCC stage I, 0/3 (insulin/noninsulin) cases showed progression versus 15/22 stable; for AJCC IIA, 0/2 progression versus 8/13 stable; IIB, 2/1 progression versus 0/10 stable; IIIB 1/1 progression versus 0/5 stable; and IV, 2/14 progression versus 0/0 stable. This accounts for a predicted 87.7% chance of disease stability for insulin positive stage 1 cases versus a 94.0% risk of progression of non insulin stage IV tumors (for model AJCC/Outcome/IHC: valid *N* = 119, *P* < 0.001).

## Discussion

The incidence of pNETs appears to be increasing according to some reports 12. It is possible that the increasing use of a number of advanced imaging modalities for different medical conditions and better pathological classification using immunohistochemical markers of neuroendocrine differentiation has contributed to this observation. The former might also explain the trend illustrated by this study of earlier stage, lower grade tumors being detected as compared to earlier reports.

The majority of reports concerning pNETs are based on small series of cases often including a mix of different types of neuroendocrine tumors, excluding the more indolent cases and often limited to patients who have undergone surgery, generating selection biases 10,12. Nevertheless, some themes have emerged from earlier studies. Younger age at diagnosis and tumor functionality has previously been described as clinical predictors of good prognosis 12. Conversely, incomplete surgical resection and tumors larger than 5 cm have been shown to be of poor prognostic impact 12. Additionally, elevated serum Chromogranin-A levels have been associated with poor prognosis 13. Recently, somatic mutations in the MEN1 and DAXX/ATRX genes have been proposed to be markers of good prognostic outcomes in sporadic pNETs 14–16. Finally, grading and staging determinants, including the presence of liver metastases, a high Ki67 labelling index, and high mitotic count, have been described as the most consistent prognostic factors of poor outcome in terms of OS 12. The extremely variable behavior of these pNETs, however, underscores the need for better markers of disease progression, particularly in lower grade, less aggressive tumors.

In this study, we focused on clinical and pathologic characteristics that impact long-term outcome of patients with pNETS. This study included cases treated surgically and nonsurgically to diminish selection bias. This analysis is limited by the fact that it includes cases from a single Endocrine Oncology referral center. This allowed inclusion of a wide range of pNET histologies, from low-grade to high-grade neuroendocrine neoplasms. In particular, our histologic examination and WHO 2010 grading were based on centralized pathology review, as opposed to large national registries from multiple centers 10,12,15. Our cases were also characterized in a systematic fashion both in terms of pathology as well as endocrine assessments.

The population described here differs from those previously described, with a relatively smaller proportion of nonfunctioning tumors and a predominance of low-grade and low-stage tumors. With these characteristics, our population better reflects current trends in the detection of pNETs: in this population, the AJCC/UICC staging proved not only to be a good predictor of OS as previously described 8,9 but also a good predictor of PFS across the spectrum of pNETs. In contrast, while the widely used WHO grading system was a helpful tool for measuring PFS, it was not a reliable predictor of OS. This unexpected result could be explained by the relatively small number of G3 tumors and also by the shorter follow-up of the G1 compared to the G2 tumors. In addition, the immunohistochemical hormone profile, independent of clinical functionality, provided an indication of less aggressive disease. This is consistent with the notion that nonfunctioning pNETs tend to behave more aggressively as indicated in an earlier study 12. However, that study was based on the earlier International Classification of Diseases for Oncology (ICD-O) classification instead of the current WHO grading or AJCC/UICC staging systems. Thus, lower or intermediate grade cases as well as hormonally active cases were not as well represented as in the current report.

Age, gender, family history and other demographics did not prove to be useful predictors of PFS in this study. Of note, patients with MEN1 syndrome in general have multiple pNETs, but this had no significant impact on PFS. This finding has important implications for clinical management of these patients, particularly those not cured by less-than-complete pancreatectomy. A more conservative approach for low-grade and/or lower stage tumors, particularly in patients with hormonally nonfunctional pNETs, is supportable by our current findings.

In this study, octreotide treatment was offered mainly to patients with clinically aggressive disease. Overall, patients receiving octreotide differed significantly from those who did not receive the medication in terms of PFS or OS. Even though widely used to control symptoms related to hormonal hyper-secretion, somatostatin analogs have uncertain clinical benefits in terms of direct antineoplastic effects. Although recent studies have shown a cytostatic effect 17–19, the impact on overall benefit in terms of OS for pNET patients remains uncertain. A recent prospective study demonstrated that octreotide LAR increased time-to-progression in patients with metastatic midgut NETs, particularly those with less than 10% liver involvement 20. It has also been reported that pNETs with a lower proliferative rate have a longer duration of cytostatic effect from somatostatin analogs 17,21. Naturally, given the retrospective nonrandomized nature of this study, we cannot definitively address this question. However, given the less than obvious impact on PFS or OS in patients with advanced stage pNETs, it would appear that octreotide does not add measurably to the management of these patients. In contrast, a direct comparison of the impact of somatostatin analogs in functional versus nonfunctional low-grade pNETs seems warranted.

In summary, our series of pNETs reflects current trends in these increasingly common endocrine tumors. Systematic clinico-pathological assessment confirms the utility of the AJCC staging and WHO 2010 grading systems in predicting PFS. Detailed endocrine tissue profiling of pNETs provides additional information of potentially important prognostic value.
